# Associations between Red Cell Polymorphisms and *Plasmodium falciparum* Infection in the Middle Belt of Ghana

**DOI:** 10.1371/journal.pone.0112868

**Published:** 2014-12-03

**Authors:** Nicholas Amoako, Kwaku Poku Asante, George Adjei, Gordon A. Awandare, Langbong Bimi, Seth Owusu-Agyei

**Affiliations:** 1 Kintampo Health Research Centre, Kintampo, Brong Ahafo Region, Ghana; 2 Infectious Diseases Research Laboratory, Department of Biochemistry, Cell and Molecular Biology. University of Ghana, Legon, Ghana; 3 Department of Animal Biology and Conservation Science, University of Ghana, Legon, Ghana; Université Pierre et Marie Curie, France

## Abstract

**Background:**

Red blood cell (RBC) polymorphisms are common in malaria endemic regions and are known to protect against severe forms of the disease. Therefore, it is important to screen for these polymorphisms in drugs or vaccines efficacy trials. This study was undertaken to evaluate associations between clinical malaria and RBC polymorphisms to assess biological interactions that may be necessary for consideration when designing clinical trials.

**Method:**

In a cross-sectional study of 341 febrile children less than five years of age, associations between clinical malaria and common RBC polymorphisms including the sickle cell gene and G6PD deficiency was evaluated between November 2008 and June 2009 in the middle belt of Ghana, Kintampo. G6PD deficiency was determined by quantitative methods whiles haemoglobin variants were determined by haemoglobin titan gel electrophoresis. Blood smears were stained with Giemsa and parasite densities were determined microscopically.

**Results:**

The prevalence of clinical malarial among the enrolled children was 31.9%. The frequency of G6PD deficiency was 19.0% and that for the haemoglobin variants were 74.7%, 14.7%, 9.1%, 0.9% respectively for HbAA, HbAC, HbAS and HbSS. In Multivariate regression analysis, children with the HbAS genotype had 79% lower risk of malaria infection compared to those with the HbAA genotypes (OR = 0.21, 95% CI: 0.06–0.73, p = 0.01). HbAC genotype was not significantly associated with malaria infection relative to the HbAA genotype (OR = 0.70, 95% CI: 0.35–1.42, p = 0.33). G6PD deficient subgroup had a marginally increased risk of malaria infection compared to the G6PD normal subgroup (OR = 1.76, 95% CI: 0.98–3.16, p = 0.06).

**Conclusion:**

These results confirm previous findings showing a protective effect of sickle cell trait on clinical malaria infection. However, G6PD deficiency was associated with a marginal increase in susceptibility to clinical malaria compared to children without G6PD deficiency.

## Introduction

It is estimated that there are about 216 million cases of malaria each year and about 655, 000 deaths worldwide [1]. Over 91% of these deaths occur in sub-Saharan Africa [1], [2]. Children under the age of five years are the most vulnerable to malaria morbidity and mortality due to their immune-naïve status [3]. However, until the age of about six months, such children are protected from malaria by maternal antibodies [4], [5]. Susceptibility to malaria increases substantially as maternal protection wanes [5], [6].

Glucose-6-phosphate dehydrogenase (G6PD) deficiency and haemoglobins S (HbS) are common genetic disorders in sub-Sahara Africa [7]. Glucose-6-phosphate dehydrogenase deficiency is a X-linked genetic disorder though most deficient people are asymptomatic [8]. Sickle cell trait (HbAS) which is clinically silent occurs when an individual inherits one gene copy of mutated haemoglobin (S) gene and one gene copy of the normal haemoglobin (A) gene. Sickle cell anaemia (HbSS) on the other hand is clinically severe and it results when two copies of mutated haemoglobin genes are inherited but both conditions are caused by haemoglobin S.

Sickle cell trait and other red blood cell−related genetic factors, such as alpha-thalassaemia, as well as metabolic abnormalities including glucose-6-phosphate dehydrogenase (G6PD) deficiency have also been associated with protection against clinical malaria [9], [10]. The degree of risk and protection against malaria conferred by genetic factors may depend on the prevalence of other co-infections.

The prevalence of G6PD deficiency in Ghana is estimated to be about 12% among pregnant women [11] and 20% G6PD prevalence has been recorded among African children with malaria [12]. About 2% of Ghanaian newborns have either sickle cell trait or disease [13] and the prevalence exceeds 25% in some areas in Africa [7]. G6PD deficiency and sickle cell disease are important causes of morbidity and mortality in Ghana [13]. The extent of risk or protection from malaria conferred by these two disorders, in the era of the changing patterns of malaria in Ghana needs to be investigated.

The Kintampo North Municipality lies within the forest-savannah transition belt of Ghana and has high levels of malaria transmission [14]. Transmission in the Kintampo area is all year round with parasite prevalence estimated to be greater than 50% in children. Annual entomological inoculation rates estimated in 2004 and repeated in 2005 were 269 and 231 infective bites per person per year respectively [15]. The area is one of the few sites in Ghana where vaccines and drugs clinical interventions aimed at reducing malaria incidence are evaluated. This study was carried out to determine the associations between G6PD deficiency and sickle cell haemoglobinopathy and clinical malaria in the Municipality. Evidence of a significant effect would provide a rationale for routine testing of red cell polymorphisms in malaria clinical trials in the area.

## Materials and Methods

### Ethical statement

Ethical approval of the study was given by the Kintampo Health Research Centre Institutional Ethics Committee. At enrolment, the study objectives, risks, benefits and procedures of the study were explained to all caregivers whose children participated in the study to seek their written informed consent. Caregivers gave written informed consent on behalf of participating children. All data collected are kept in locked cabinets to ensure confidentiality and participants’ identity and records were anonymized prior to analysis.

### Subjects, Design and Sample Collection

This was a cross-sectional study, carried out at the Kintampo North Municipal Hospital in the Brong Ahafo Region of Ghana from November 2008 to June 2009. Children less than five years of age who reported of fever (i.e. measured axillary temperature ≧37.5°C or reported fever in 48 hours prior to hospital attendance) and without symptoms of severe illness were enrolled. All enrolled children were residents of the Kintampo North Municipality as per study inclusion criteria where malaria transmission intensity is as high as 269 infective bites/person/year and malaria parasite prevalence is >50% [14], [15].

Individual written informed consent was obtained from all caregivers prior to enrolment. A study clinician initially took clinical history and carried out a physical examination on all children irrespective of their disease condition, after which blood samples were taken to test for malaria and host genotypes including sickle cell. Recruitment into the study covered both the dry (November) and rainy (June) seasons as the causes of fevers are not the same throughout the year and each child was seen once at the outpatient department (OPD) without any follow up visit. Any child diagnosed as having clinical malaria, defined as fever (i.e. measured axillary temperature ≧37.5°C or reported fever in 48 hours prior to hospital attendance) plus a positive malaria blood smear, was treated with artesunate/amodiaquine; other diseases diagnosed were treated according to the Ghana Health Service (GHS) standard treatment guidelines. Information on socio-demographic background was obtained through the use of a short questionnaire. About 0.5 ml of finger-prick capillary blood was collected into EDTAK2 anticoagulant tubes and used for the laboratory analysis at the Kintampo Health Research Centre (KHRC) Clinical Laboratory. All blood samples were obtained prior to treatment with artesunate/amodiaquine.

### Laboratory Procedures

#### G6PD activity

G6PD status was determined by the Randox quantitative method [16], [17]. Briefly, 0.2 ml of whole EDTA blood was washed three times with 2 ml aliquots of 0.9% sodium chloride (NaCl) solution, centrifuged after each wash for 10 minutes at 3000rpm. The G6PD activity was determined using the washed erythrocytes and the test method performed as described in the manufacturer’s instruction manual (Randox Laboratory UK, BT29 4 QY). G6PD activities were expressed in U/g Hb using the respective haemoglobin (g/dl) concentrations estimated with Micro 60 haematology analyzer (Horiba ABX, Montpellier, France).

The Randox quantitative method classifies the G6PD deficiency status of the participants using a cut-off of 4.1 U/g Hb. All participants who measured enzyme activity <4.1 U/g Hb were classified as “Deficient” while those with enzyme activity at 4.1 U/g Hb and above were “Normal”. There was no classification for partial deficiency. Two levels of quality controls, deficient (catalogue no. PD2617) and normal levels (catalogue no. PD2618) were analyzed before the analysis of test samples.

#### Haemoglobin concentration

Haemoglobin levels were determined using an ABX Micros 60 Haematology analyzer (Horiba ABX, Montpellier, France). Scheduled external quality assessment programme and daily internal quality controls were followed as quality measures [18].

#### Haemoglobin Typing

The Helena electrophoresis system (Helena Biosciences Europe, Queensway South, Gateshead Tyne and Wear NE II OSD) was used to electrophorese haemolysates on Titan III cellulose acetate plates. The haemolysate was prepared by mixing 1 part of whole blood to 3 parts Helena haemolysate reagent (0.005 M EDTA with 0.01% potassium cyanide). Electrophoresis was performed at 350 V for 25 minutes in a tris-EDTA/boric acid buffer (pH 8.4, ionic strength 0.035; Helena). The electrophoretic procedure was performed as recommended by the manufacturer. Helena Hemo quality controls provided and performed daily were used as a maker for band identification.

#### Malaria parasitaemia

Thin and thick blood smears were prepared; the thick film was fixed in methanol after drying and both thin/thick films stained with 10% Giemsa. The parasites were counted against 200 leukocytes and then extrapolated to parasites per microlitre of blood using the absolute leucocytes counts [14]. At least one hundred thick film fields were examined before assigning a negative malaria diagnosis. Two expert microscopists at the clinical laboratory of Kintampo Health Research Centre who read each blood slide were blinded from each other’s reading. All discordant readings were re-read by a third microscopist who was blinded from the previous results.

### Data Management and Statistical Analysis

All the data were double-entered and verified for consistency using Microsoft Access software version 2007. Final dataset was analyzed using STATA statistical software (Version 11; StataCorp. TX USA). Proportions of categorical variables such as bednet use or antimalarial use were compared using the χ*^2^*-test and means of continuous and normally distributed variables such as haemoglobin level were compared using t*-*test (two-group comparison) and one way ANOVA (three-group comparison). Statistical significance was set at *p<*0.05. Using a forward selection method, a logistic regression model controlling for potential confounding variables such as antimalarial use and bed net use were used to investigate the associations of G6PD and sickling status on clinical malaria. High density malaria parasitaemia (HDP) was defined as malaria parasites ≧10,000/µL of blood and severe anaemia was defined as haemoglobin concentration level less than 6.0 g/dL.

## Results

### Demographic and clinical data

A total of 364 children were approached to be consented. Ninety-four percent (341/364) were enrolled; 75% of the children enrolled into the study were above 11 months of age with remaining 25% either aged 11 months or below (Table 1). About 53% of the children were males. Twenty-five percent of children had been transfused with blood previously in more than 6 months prior to enrolment as a result of earlier illness but children with recent blood transfusion(6 months or less) were not enrolled (Table 1). Insecticide treated bed net (ITN) use was 69.2% among the children enrolled (Table 1).

**Table 1:** Baseline characteristic of the study population.

Characteristic	n(%)
**Age group**	
0–11 mo*	84(24.6)
12–59	257(75.4)
**Sex**	
male	180(52.8)
female	161(47.2)
**Siblings**	
alive	321(94.1)
dead	20(5.9)
**Blood transfusion**	
**ever transfused**	
Transfusion Intervals**	84(24.6)
8–9 mo*	24(7.1)
10–16 mo	60(17.5)
**never transfused**	257(75.4)
**No. of times transfused**	
once	52(61.9)
twice	20(23.8)
more than twice	12(4.3)
**Bed net use**	
yes	234(69.2)
no	107(30.8)

*mo = age in months, n = total number of children, % = percentage of children.

**Interval of transfusion (in months) before enrolment.

Basic profiles of the study children as captured by questionnaire have been summarize. The age group, sex and information of siblings and whether or not they have ever received blood transfusion and level of bed net usage have been tabulated.

### Frequency of Red Cell Polymorphisms

About one-fifth (19%) of the children were G6PD deficient (Table 2); 55.4% (36) of G6PD deficient children were males. HbAS and HbAC genotypes were present in 9.1% (31) and 14.7% (50) of 341 children respectively (Table 2). HbSC (1), HbCC (1) and HbSS (3) genotypes occurred in less than 2% and were excluded from the regression analysis. Twenty percent (50/255) of the children with HbAA also had G6PD deficiency whilst 22% (11/50) of those with HbAC had G6PD deficiency but only 13% (4/31) of HbAS individuals had G6PD deficiency.

**Table 2:** The prevalence of red blood cell polymorphisms.

Red Cell Polymorphisms		n (%)
**Haemoglobin variants**	**HbAA**	**255 (74.7)**
	HbAS	31 (9.1)
	HbAC	50 (14.7)
	HbCC	1 (0.3)
	HbSC	1 (0.3)
	HbSS	3 (0.9)
**G6PD variants**	Normal	276 (80.9)
	Deficient	65 (19.1)

n = total number of children, % = percentage of children.

Children with different haemoglobin types as determined by electrophoresis have been presented in terms of number (n) and percentages (%) to show their prevalence. HbAA = homozygous wild type genotype, HbAS = Heterozygote sickle cell haemoglobin, HbAC = heterozygote haemoglobin C, HbSC = heterozygote haemoglobin S and C, HbCC = homozygote haemoglobin C, HbSS = homozygote haemoglobin S. G6PD variants has been determined quantitatively using commercial kit from Randox Laboratory.

### Haemoglobin levels

**Figure 1:**
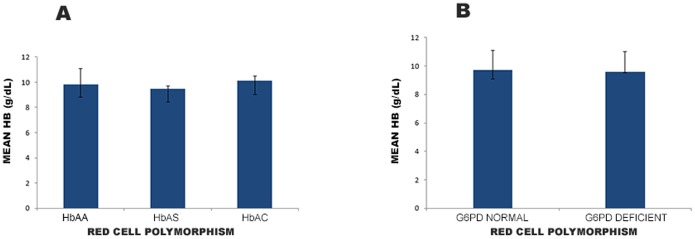
Mean Haemoglobin levels for children with different red cell polymorphisms. About 0.5 ml of capillary blood by finger prick was collected into EDTAK2 anticoagulant tubes for each child and after performing the daily quality controls, the samples run on ABX Micro60 Haematology analyzer (ABX-Horiba, France) to determine haemoglobin levels of children with different red cell polymorphism. Mean haemoglobin levels (mHb) were calculated for the different red cell polymorphisms. mHb for the Haemoglobin variants (**A**) and G6PD variants (**B**) are indicated by the bars and their confidence intervals (CI) indicated as lines.

Children with HbAC genotype had the highest mean haemoglobin (mHb) of 10.1 g/dL (95% CI: 9.0–10.5) and HbAS genotype had the lowest of 9.5 g/dL (95% CI: 8.4–9.7) (Figure 1a). G6PD normal and deficient individuals had mHb of 9.7 g/dL (95% CI: 9.1–11.1) and 9.6 g/dL (95% CI: 9.5–11.0) respectively (Figure 1b). The mHb for G6PD deficient males and females were not significantly different (p = 0.40) but the mHb was significantly different among male and female participants with G6PD normal (p = 006). The proportion of severe anaemia (haemoglobin concentration <6 g/dL) for children in the entire cohort was 2.6% (9/341). About 4.6% (3/65) of G6PD deficiency children had anaemia but no child with the sickle cell trait had haemoglobin level less than 6.0 g/dL.

### Red cell polymorphisms and clinical malaria

The overall prevalence of clinical malaria in the entire cohort of children was 31.9% (107/336). Univariate and multivariate models for clinical malaria infection defined as the presence of fever and parasites have been summarized in Table 3. In the multivariate regression analysis, children with HbAS genotypes were 79% less likely to have clinical malaria as compared to those with HbAA genotype (adjusted OR = 0.21, 95% CI: 0.06–0.73, p = 0.01). The risk of clinical malaria was statistically not different among children with HbAC genotype relative to those with HbAA genotype (adjusted OR = 0.70, 95% CI: 0.35–1.42, p = 0.33). The G6PD deficient children were 1.76 times more likely to contract malaria as compared to the G6PD normal subgroup, although this association was not significant (adjusted OR = 1.76, 95% CI: 0.98–3.16, p = 0.06).

**Table 3:** Association between the red cell polymorphisms and clinical malaria.

Red CellPolymorphisms		Children withclinical malaria[n(%)]	Children withoutclinical malaria[n(%)]	Univariate(unadjusted)Odds ratio(95% CI)	p-value	Multivariate(adjusted)Odds ratio(95% CI)	p-value
**Haemoglobin** **variants**	HbAA	90(84.1)	165(72.1)	1	–	1	–
	HbAC	14(13.1)	36(15.7)	0.71(0.37–1.39)	0.321*	0.70(0.35–1.42)	0.356*
	HbAS	3(2.8)	28(12.2)	0.20(0.06–0.66)	0.009*	0.21(0.06–0.73)	0.014*
**G6PD** **variants**	Normal	80(74.8)	191(83.4)	1	–	1	–
	Deficient	27(25.2)	38(16.6)	1.70(0.97–2.96)	0.063	1.76(0.98–3.16)	0.060
**Bed net** **use**	Yes	72(67.9)	163(70.5)	1	–	1	–
	No	34(32.1)	67(29.5)	1.13(0.69–1.86)	0.636	1.17(0.69–1.99)	0.562
**Antimalarial** **use**	Yes	23(21.5)	91(39.7)	1	–	1	–
	No	84(78.5)	138(60.3)	2.41(1.42–4.10)	0.001	2.70(1.52–4.64)	0.001
**Gender**	Male	56(52.3)	122(53.3)	1	–	1	–
	Female	51(47.7)	107(46.7)	1.04(0.66–1.64)	−0.872	1.09(0.67–1.78)	−0.725
**Age(months)**	0−11	16(15.0)	68(29.7)	1	–	1	–
	12−59	91(85.00)	161(70.3)	2.40(1.32–4.39)	−0.004	(2.44(1.30–4.56)	−0.005
**Male G6PD** **deficient**	NormalDeficient	44(78.6)	98(80.3)	1	–	1	–
	NormalDeficient	12(21.4)	24(19.7)	1.11(0.51–2.43)	−0.787	1.20(0.53–2.71)	−0.657
**Female G6PD** **deficient**		36(70.6)	93(86.9)	1	–	1	–
		15(29.4)	14(13.1)	2.77(1.22–6.31)	−0.015	2.68(1.10–6.52)	−0.031

*Likelihood ratio p-value <0.01.

n = number of children % = percentage of children CI = Confidence Interval.

Red cell polymorphisms were stratified according to the presence or absence of clinical malaria for analysis. Univariate and multivariate logistic regression analyses were also performed. The odds ratio (OR) and p-values have been calculated to determine the level of significance of association between the red cell polymorphisms and clinical malaria. Subgroup analysis for HbSC, HbCC and HbSS was not included due to small numbers obtained.

The risk of malaria in children who used bed nets was not significantly different from those who did not use bed nets (adjusted OR = 1.17, 95% CI: 0.69–1.99, p = 0.56). Children who did not use antimalarial had higher odds of contracting clinical malaria as compared to children who used antimalarial (adjusted OR = 2.70, 95% CI: 1.52–4.64, p = 0.001). Children aged 12–59 months were more likely to have malaria as compared to children aged 0–11 months (adjusted OR = 2.44, 95% CI: 1.30–4.56, p = 0.01). Among male children, the G6PD deficient children had higher odds of contracting malaria but this was not statistically significant (adjusted OR = 1.20, 95% CI: 0.53–2.71, p = 0.657). Similarly among females, children who were G6PD deficient were more likely to contract malaria as compared to G6PD normal children (adjusted OR = 2.68, 95% CI: 1.10–6.52, p = 0.031)(Table 3).

### Red cell polymorphism and malaria parasite infection

**Figure 2:**
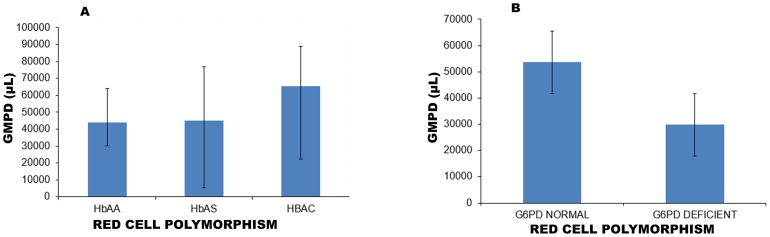
Geometric mean parasite density (GMPD) of children with the different red cell polymorphisms. Peripheral blood collected into EDTAK2 anticoagulant was used to prepare both thick and thin blood smears. Giemsa stained thick blood films were used for parasitological assessment and parasite densities were calculated using the absolute leucocytes counts. The parasitaemia per µl of blood was calculated by using the WHO (1996) formula:  = (Number of parasites counted/WBC counted) × absolute WBC count/µL of participant. Parasite densities are reported as geometric means for parasitaemic children with (**A**) showing haemoglobin variants, HbAA (n = 167), HbAS (n = 10), HbAC (n = 37) and (**B**) G6PD normal (n = 182), G6PD deficient (n = 37) genotypes. Thus bars represent GMPD of individual red cell polymorphisms and their 95% confidence intervals represented as line.

The geometric mean parasite density (GMPD) observed among children with the red cell polymorphism variants were: HbAC [65451/µL (95% CI: 22424–91039)], HbAS [45045/µL; (95% CI: 5384–76899)], HbAA [43954/µL (95% CI: 30154–64071)] (Figure 2a). There was no evidence of a significant relationship between these red cell polymorphism and GMPD (one way ANOVA Bartlett’s test Chi^2^ = 1.232, p = 0.54).

GMPD among G6PD deficient [29776/µL (95% CI: 14858–59672)] and G6PD normal [53629/µL; (95% CI: 35931–80044)] children were similar (one way ANOVA Bartlett’s test Chi^2^ = 0.162, p = 0.69) (Figure 2b).

**Figure 3:**
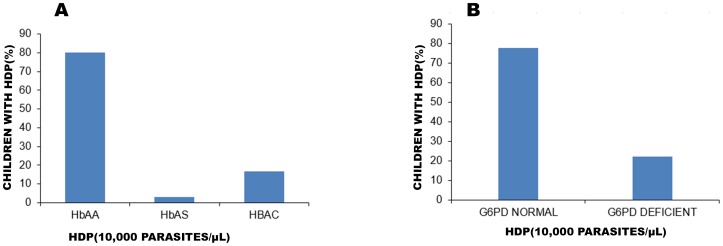
Proportion of children with high density parasite (HDP) in the red cell polymorphisms. Children with high density parasite (HDP) defined as ≧10,000 parasites/µL of blood was classified according to the red cell polymorphisms. This cut off value was used because of the likelihood of a clinical attack when blood-stage parasites density exceeds 10,000 parasites/µL in endemic regions. Thick blood films were stained with Giemsa and the numbers of malaria parasites were counted against leukocytes. The microscopist counted fields containing 200 leukocytes. If <10 parasites were observed, the counting continued up to 500 leukocytes. Where microscopists did the parasite counts in the thin film (against 2,000 red blood cells) as a result of heavy parasitaemia (greater or equal to 100 parasites per thick smear high power field), parasites counted were recalculated with 200 WBC. Parasite densities were calculated using absolute leukocytes/L of blood. (**A**), Bars indicate percentage of children with HDP in the haemoglobin variants subgroup and (**B**) bars showing HDP in the G6PD variants subgroup.

High-density parasitaemia–HDP (parasites ≧10,000/µL of blood) was observed in 16.8% (22/131) of HbAC and 3.1% (4/131) among the HbAS when compared to 80.2% (105/131) of the HbAA subgroup (P = 0.007) (Figure 3a). G6PD normal children with HDP was 77.9% (102/131) and that for the G6PD deficient subgroup was 22.1% (29/131) (Figure 3b). However, the G6PD status was not associated with high-density status (p = 0.30).

## Discussion

Our results have demonstrated a reduction in malaria risk in children with the sickle cell trait, an association that remained statistically significant in multivariate regression analysis after controlling for bed net and antimalarial drug use. Protection associated with HbAC was less significant as compared to HbAS genotype. G6PD deficiency was however associated with marginal susceptibility with clinical malaria.

Many studies globally [9], [19], have confirmed the protective role of sickle cell trait in mild malaria disease, and so the relationship to clinical malaria found in this study is not very surprising. The results obtained in this study support the hypothesis that haemoglobin S offers selective advantage to carriers of HbAS against clinical malaria (OR = 0.21, 95% CI: 0.06–0.73, p = 0.014), consistent with similar studies in Kenya [9] and Gambia [20]. A number of possible biochemical and immune-mediated mechanisms have been proposed to be responsible for the observed protection in HbAS individuals [21]. This include enhanced phagocytosis by monocytes in HbAS infected red blood cells compared to Infected HbAA and other cells due to increased presentation of opsonins including membrane bound IgG and hemichromes [22]. The sickle cell trait is also reported to offer protection in severe malaria through mechanisms such as impaired cytoadherence and rosette forming [21], [23] and translocation of host microRNA into parasite mRNA to reduce intra-erythrocytes growth [24].

Studies conducted in Nigeria [25] and Mali [26] that assessed the role of HbAC and other haemoglobinopathies with respect to susceptibility to malaria have documented contrasting results. Whilst the study in Nigeria [25] and one study in Mali [26] indicated lack of protection of HbAC against clinical malaria, another Malian study [27] showed evidence of HbAC protection against clinical malaria. Our result showed a reduced malaria risk for HbAC phenotype in support of the second Malian study [27] although this association was not statistically significant. In a case-control study in Burkina Faso [28], HbAC was associated with 29% reduction in risk of clinical malaria and 93% reduction in HbCC homozygote. The level of protection by HbAC in the Burkina Faso study (OR 0.71; 95% CI: 0.58, 0.87) is comparable to the results obtained in this study (OR = 0.70; 95% CI: 0.35–1.42, p = 0.326). Inconsistencies in malaria-protective effect in many of the structural haemoglobin variants may partly be a result of differences in study designs and data analysis strategies used in the various investigations [29].

Nineteen percent (19%) of our study participants had G6PD deficiency, as determined phenotypically. This is consistent with the prevalence of G6PD deficiency assessed as part of a Phase III antimalarial drug trial in six African countries including Ghana [12].

The protective role of G6PD deficiency in clinical malaria has also produced conflicting results among various studies [25], [30]. Ruwende *et al*. found that G6PD deficiency was associated with a 46–58% reduction in risk of malaria in African children [31]. In some instances, G6PD deficient individuals infected with *Plasmodium falciparum* were less ill than non–G6PD-deficient individuals [31]. The explanation could be that *Plasmodium falciparum* infection is usually not lethal in G6PD deficient individuals [30] possibly because *Plasmodium* parasites can proliferate less efficiently in their erythrocytes [32].

However it is still uncertain whether this advantage applies only to individuals who are totally deficient of the G6PD enzyme. Guindo *et al*. (2007) found out that partially deficient females suffer the same morbidity and mortality of *Plasmodium falciparum* infection as non-deficient patients [33]. Mockenhaupt *et al*. (2003) also found fewer *Plasmodium falciparum* infections in pregnant women with partial G6PD deficiency [11]. In our study, the prevalence of *Plasmodium falciparum* infection was 60% among the G6PD deficient subgroup and 42% among G6PD normal subgroup with a weak evidence to support a statistical difference (p = 0.03).

One of the most frequently cited field studies of the relationship between G6PD deficiency and *falciparum* malaria is that of Bienzle *et al*. in Nigeria where 702 children aged 9 months to 6 years presenting at hospital with acute febrile illness were assessed [34]. In that study, 66% of the children with G6PD deficiency were found to have *P. falciparum* parasitaemia which is quite similar to what has been observed in our study population [34].

Nevertheless variations in our findings with others reported elsewhere could have resulted from differences in methods and populations involved. We were unable to screen for additional co-infections, such as HIV-1 and bacteraemia, believed to influence the course of malaria infection [35]. Other studies have been performed as a community-based longitudinal investigation in which both malaria morbidity and all-cause mortality were assessed in those children [10]. It is possible that the children investigated in a cross-sectional study like ours, at the hospital with their first episode of malaria may be clinically very distinct from those children residing in the community that are presumably less ill and hence such differences in results. We also think that G6PD deficient individuals with malaria infection could easily haemolyse and thus, be brought to hospital for care and this could account for high *Plasmodium* parasitisation in G6PD deficient children.

Unlike insecticide treated bed net usage, children who had not been treated with antimalarial drug were more likely to have clinical malaria. Therefore anti-malaria use both as a preventive or curative treatment is likely to reduce prevalence of the clinical malaria especially in children less than 5 years.

We could not examine the effects of three genotypes (HbCC, HbSS, and HbSC) due to their small proportion and the statistical limitations associated. This suggests the need for a bigger study in the area and under different levels of malaria transmission to better understand the patterns of protection or risk of malaria that these other phenotypes may confer in this population.

We acknowledge some limitations of this study. First, we did not investigate the role of thalassaemia in clinical malaria which could impact on the risk estimation in this study. The prevalence of thalassaemia particularly α-thalassaemia is more than 80% in parts of Africa, Southeast Asia and Melanesia [36]. Coinheritance of the α-thalassemia, among individuals with sickle cell disease appears to be protective against some sickle cell complications, such as acute chest syndrome, anaemia, and cerebrovascular accidents [37]. However, α-thalassaemia interaction with HbAS has been found to nullify the protective effect of both on malaria [38]. Secondly, the risk of malaria exposure at the individual participant level was not assessed in this study. Malaria transmission intensity varies within microecological zones [39], [40] and children who did not have malaria may not have been exposed to malaria. However, in the study area, the force of infection is extremely high (269 infective bites/person/year) [14] with little variance between communities [15] and the incidence of clinical malaria among children is high [41]. Thirdly, the risk of malaria infection which is relatively a common outcome in a malaria endemic area has been estimated using odds ratios as an approximation for risk ratios. This approach may have overestimated the odds ratios realised here.

## Conclusion

From the perspective of protecting individuals from clinical malaria, HbAS genotype has showed a clear advantage and superiority as compared with HbAC and G6PD deficient genotypes in our study population. The biological interactions between HbAS and clinical malaria has suggested the need to critically look at HbAS as an innate host factor in randomized trials or observational studies for detecting the effect of interventions, especially in areas of high HbAS prevalence.

As millions of people are exposed to malaria annually, there is the need to continually explore all possible associations that red cell polymorphisms may have with disease susceptibility or protection when designing malaria intervention programs.

## Supporting Information

Table S1 Final dataset for public repository (in STATA file).
(DTA)
